# From Vesicles to Protocells: The Roles of Amphiphilic Molecules

**DOI:** 10.3390/life5010651

**Published:** 2015-03-02

**Authors:** Yuka Sakuma, Masayuki Imai

**Affiliations:** Department of Physics, Tohoku University, Aoba, Sendai 980-8578, Japan; E-Mail: sakuma@bio.phys.tohoku.ac.jp

**Keywords:** protocell, vesicle, deformation, spontaneous curvature, phase separation, adhesion, pore formation, self-reproduction

## Abstract

It is very challenging to construct protocells from molecular assemblies. An important step in this challenge is the achievement of vesicle dynamics that are relevant to cellular functions, such as membrane trafficking and self-reproduction, using amphiphilic molecules. Soft matter physics will play an important role in the development of vesicles that have these functions. Here, we show that simple binary phospholipid vesicles have the potential to reproduce the relevant functions of adhesion, pore formation and self-reproduction of vesicles, by coupling the lipid geometries (spontaneous curvatures) and the phase separation. This achievement will elucidate the pathway from molecular assembly to cellular life.

## 1. Introduction: A Scenario from Molecular Assembly to Protocell

### 1.1. Formation of Vesicle

On the primitive Earth, numerous chemical reactions of organic compounds produced the ingredients of life. Of these substances, amphiphilic molecules might be the first player in the evolution from molecular assembly to cellular life [1,2]. When the concentration of synthesized amphiphilic molecules is very low, the molecules diffuse in water individually. As the concentration increases, amphiphilic molecules start to assemble due to hydrophobic interactions [3,4]. The formation of molecular assembly, as measured by the critical aggregation concentration (c.a.c.), strongly depends on the lipid chemical structure. Primitive amphiphilic molecules, fatty acids, have a high c.a.c. of 10^−1^–10^−3^ M [5], whereas modern amphiphilic molecules, phospholipids, have a low c.a.c of ~10^−^^10^ M [6]. Thus, the formation of molecular assemblies requires that primitive amphiphilic molecules are concentrated in water.

**Figure 1:**
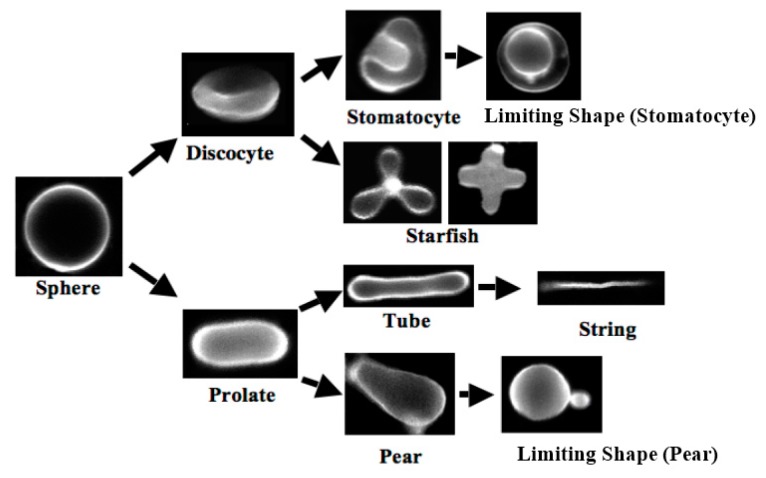
Shape deformation pathways of the one-phase giant unilamellar vesicles (GUVs) under a constant osmotic pressure difference (taken from [8]).

The shape of the assembly is determined by the geometry of the amphiphilic molecule [3]. Molecules that have a cylindrical shape prefer to form flat bilayers. To avoid direct contact between water and the edge of the bilayer, the bilayer forms a closed membrane, *i.e.*, a vesicle. A unique feature is that vesicle shape can be modulated by changing the external conditions. For example, when solute is added to the external medium, the vesicle exhibits a parade of shape transitions through the excess area produced by the osmotic pressure difference. The shape deformation pathways of the one-phase giant unilamellar vesicles (GUVs) under a constant osmotic pressure difference are shown in Figure 1 [7,8]. By adding solute, the shape of spherical GUV starts to fluctuate and then transforms to prolate or discocyte [9]. A further increase in the excess area causes bifurcations from prolate (discocyte) shapes to tubes (stomatocyte shape) and pears (starfish). The observed shape deformations are well described by the area difference elasticity (ADE) theory [10,11,12]. In the ADE theory, the vesicle shape is determined from the minimization of the total elastic energy, *F*_t_, given by: 

(1) $$F_{t}=F_{b}+F_{ADE}$$

(2) $$F_{b}=\frac{\kappa }{2}\oint {\left(2H-C_{0}\right)}^{2}dA+\kappa_{G}\oint K\;dA$$

(3) $$F_{ADE}=\frac{\kappa_{r}}{2Ad^{2}}{\left(\Delta A-\Delta A_{0}\right)}^{2}$$

The first term, *F*_b_, is the bending energy of the membrane, where *A* is the membrane area, κ and κ_G_ are the local bending and the Gaussian bending rigidity, respectively, *C*_0_ is the spontaneous curvature and *H* and *K* are the mean curvature and the Gaussian curvature, respectively. For a vesicle having a closed surface, the integral of the second term of Equation (2) depends only on the topology (number of handles *g*) of the vesicle, *i.e.*, ∮K dA=4π(1−g), due to the Gauss–Bonnet theorem [12]. Then, we can discard this second term as long as the vesicle maintains a fixed topology. The area difference elasticity term, *F*_ADE_, is associated with the relative stretching of the monolayers in the bilayer, where κ_r_ is the non-local bending modulus and *d* is the distance between the monolayer’s neutral planes. The bilayer has an intrinsic area difference given by $\Delta A_{0}=(N^{+}-N^{-})a$, where *a* is the equilibrium area per membrane molecule and *N*^+^ and *N*^−^ are the numbers of molecules in the outer and inner leaflets, respectively. The geometrical area difference $\Delta A=A^{+}-A^{-}$, where *A*^+^ and *A*^−^ are the areas of the outer and inner leaflets, is expressed by an integral over the mean curvature as ΔA=2d∮H dA. If the monolayer area difference Δ*A* deviates from the intrinsic value Δ*A*_0_, the monolayers are stretched relative to one another, and the elastic energy is expressed by Equation (3). Minimization of the total energy for a given area *A* and volume *V* gives the vesicle shape. In this ADE model, the vesicle shape having *C*_0_ = 0 is determined by two geometrical parameters, the reduced volume being expressed as:

(4) $$v=\frac{V}{4\pi R_{s}^{3}/3}$$

where $R_{s}=\sqrt{A/4\pi }$ and the reduced intrinsic area difference: 

(5) $$\Delta a_{0}=\frac{\Delta A_{0}}{8\pi dR_{s}}$$

**Figure 2:**
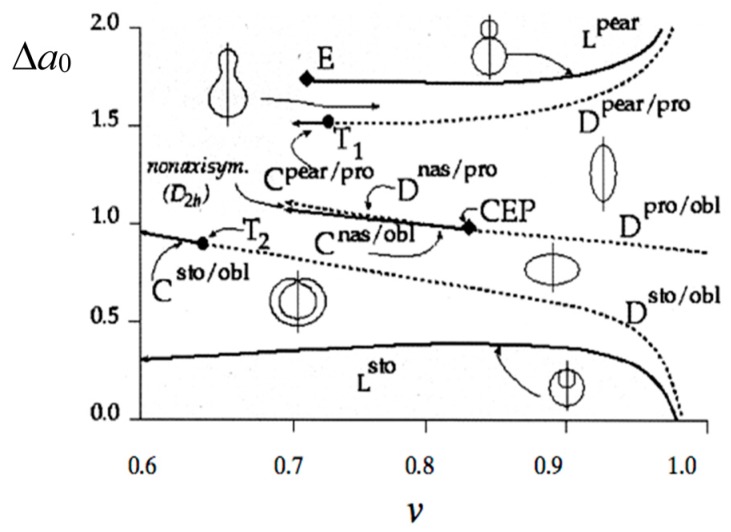
Phase diagram of the area difference elasticity (ADE) model. Characteristic equilibrium shapes, pears, prolates (pro), oblates (obl), stomatocytes (sto) and elliptical non-axisymmetric shapes (nas), are illustrated for each phase and for the two limiting lines (L^pear^ and L^sto^), where two spheres are connected by a very thin neck. First order discontinuous transitions (D) are shown as dashed lines; second order continuous transitions are shown as full lines. At the special point E, the radii of the two spheres of the limiting pear shape become equal. Special critical points are indicated by T_1_, T_2_ and critical end point CEP (taken from [13]).

The vesicle shapes obtained by the total energy minimization are mapped in a *v −* Δ*a*_0_ phase diagram, as shown in Figure 2 [13]. This ADE theory is quantitatively supported by vesicle fluctuation analysis [14] and a 3D analysis of vesicle shapes [15]. Thus, to attain the vesicle deformation relevant to a protocell, we should control the reduced volume and the reduced intrinsic area difference by changing the external environment.

### 1.2. From Vesicle to Protocell

The formation of a vesicle having the ability to change its shape in response to external stimuli is considered to be an important step in the transition from inanimate organic compounds toward cellular life. Actually, the amphiphilic molecules present in the Murchison meteorite form membranous vesicles when exposed to dilute aqueous solution [16,17]. To guide the way from vesicle to cellular life, the concept of the protocell has been introduced. A protocell is a virtual cell having minimal functions to support life. This concept has been reviewed in many books [18,19,20,21,22]. The protocell is composed of three fundamental components, a metabolism that extracts usable energy and resources from the environment, genes that chemically exert informational control of living functionalities and a container that keeps them all together. The vesicle is a suitable container for the protocell, which exhibits self-reproduction cooperating with the metabolic system. In the vesicle, amphiphilic molecules that are synthesized from ingredients form new vesicles. The information molecules control the reaction network of the metabolism. The coupling between the self-reproducing vesicle and the self-replicating information molecule is a feature of the protocell [23].

**Figure 3:**
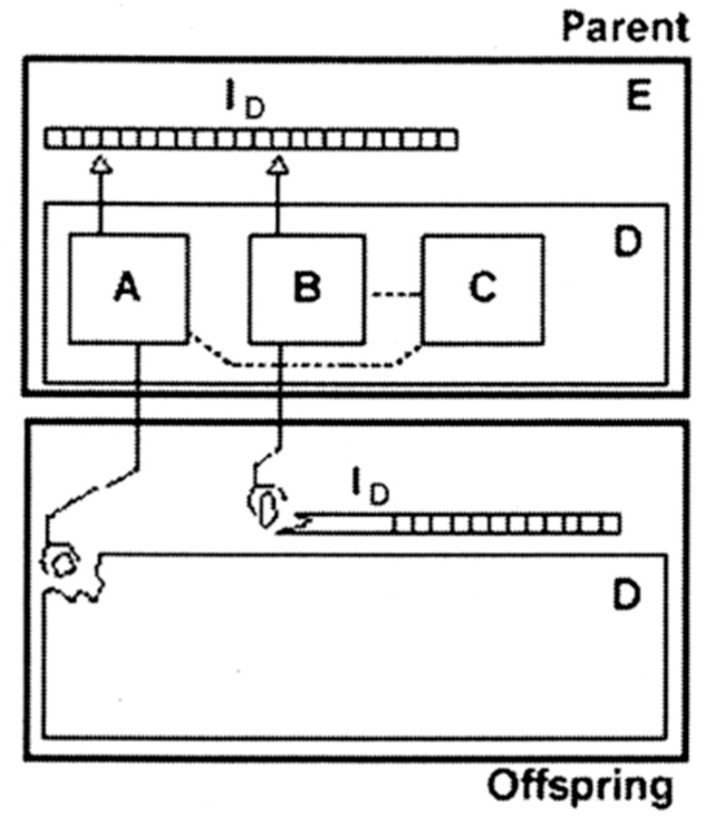
A self-reproducing automaton based on Von Neumann’s logic (taken from [25]). I_D_, instruction tape; A, universal constructor; B, copier; C, controller; D, and self-reproducing automaton; E.

It is worthwhile to note that the protocell concept agrees with an artificial automaton proposed by Von Neumann [24,25]. This automaton is composed of the information part (I_D_) and the construction part (D), as shown in Figure 3. The information part (corresponding to the gene in the protocell) is defined as a tape, which is both a code and a program. The construction part (container) contains three components, A, B and C (the reaction network of metabolism). The universal constructor (A) constructs the offspring part (D) according to the instructions encoded in the tape (I_D_), whereas the copier (B) replicates the instruction tape (I_D_). The controller (C) controls the operations of the constructor and the copier. The new automaton follows the same scheme to self-reproduce. Thus, the gene, the metabolism and the container are elementary units for an autonomous system.

**Figure 4:**
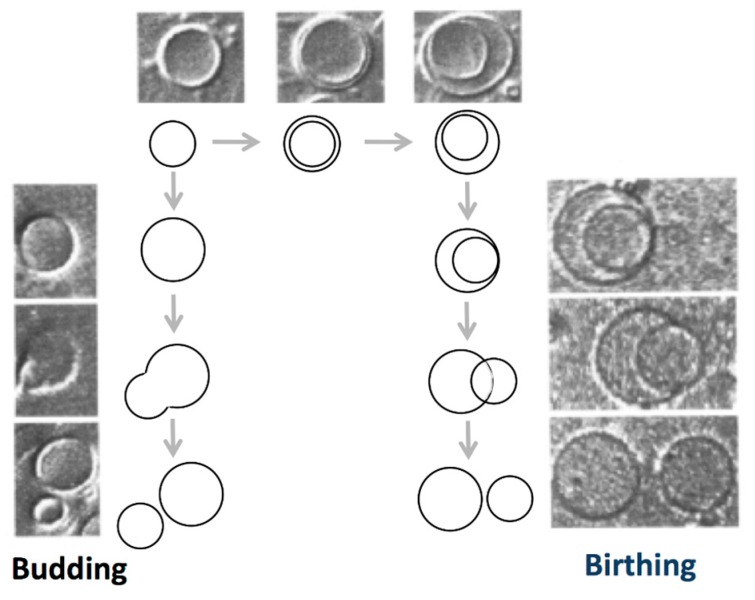
Two self-reproduction processes observed in oleic acid/oleate giant vesicles following oleic anhydride hydrolysis: “budding” and “birthing” (taken from [32]).

A step from vesicle toward protocell might be to develop self-reproducing vesicles coupled with the metabolic system. This approach was summarized in review articles [1,2,26,27,28,29,30]. In pioneering works, self-reproduction of vesicles was reported for an oleic acid/oleate giant vesicle system [31,32]. In the case of fatty acid vesicles, the morphology of the self-assembly is governed by the association of the carboxyl group, and vesicle formation is observed in the restricted pH region, where approximately half of the carboxyl groups are ionized [26,33]. Upon the addition of a droplet of oleic anhydride to an oleic acid/oleate giant vesicle suspension, the anhydride molecules are hydrolyzed to oleic acid/oleate within the bilayer of the vesicle. The vesicles supplemented with the oleic acid/oleate molecules display two self-reproduction processes: a budding pathway, where the mother vesicle deforms to a pear-like shape and then divides into two vesicles, and a birthing pathway, where the mother vesicle forms an inclusion vesicle that is then expelled from the mother vesicle, as shown in Figure 4.

**Figure 5:**
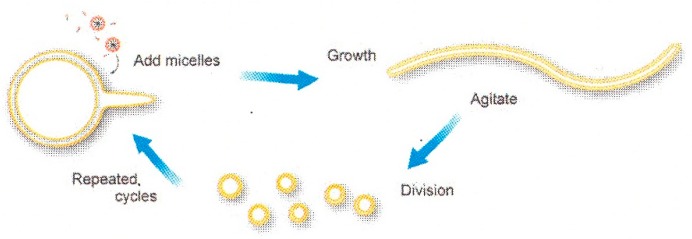
Schematic diagram of cyclic multilamellar vesicle growth and division (taken from [34]).

Another potential mechanism consists of the cyclic self-reproduction of fatty acid vesicles [34,35]. This self-reproduction process starts from spherical multilamellar oleic acid/oleate vesicles, as shown in Figure 5. By addition of oleate micelles, thin tails begin to protrude from the mother vesicle, and finally, the initially spherical vesicles completely transform into long, thread-like vesicles. By applying mild agitation (shear force), the thread-like vesicles divided into multiple smaller, spherical, multilamellar daughter vesicles. Upon further micelle addition, the daughter vesicles grow to the sphere-tail intermediate stage, and the growth-division cycle continues in a cyclic manner.

**Figure 6:**
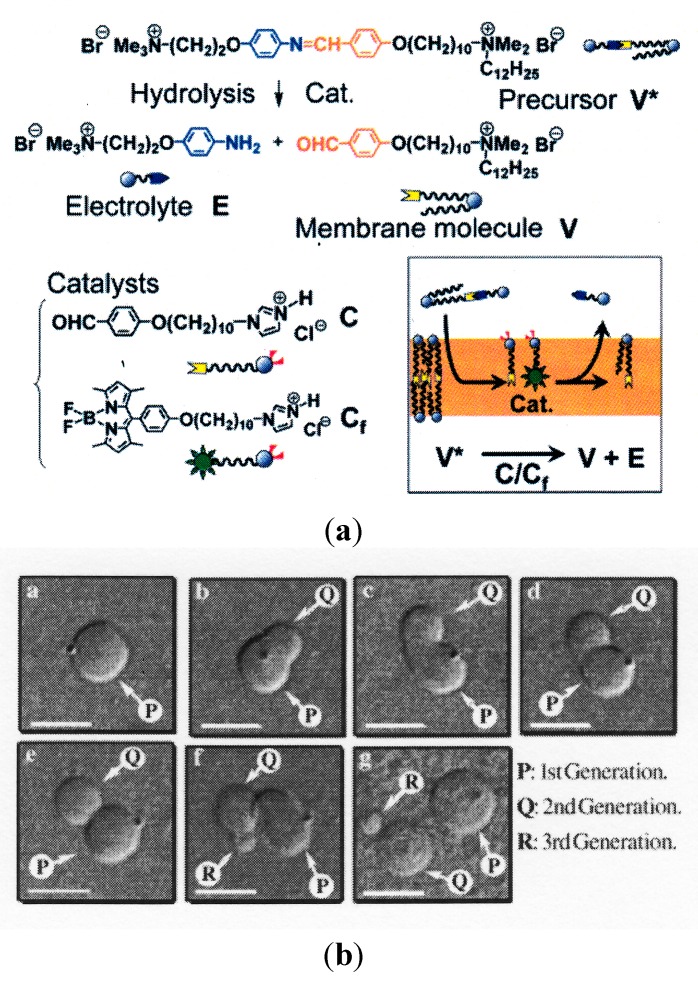
(**A**) Schematic representation of the self-reproduction of giant multilamellar vesicles (GMVs). The membrane molecule, V, and electrolyte, E, are formed by the hydrolysis of the membrane precursor, V*, in the presence of the catalyst, C, and the fluorescence probe, C_f_, which are anchored within the vesicular membrane (panel at the bottom right) (taken from [37]). Cat.: Catalysts. (**B**) Morphological changes in a GMV composed of V and 10 mol% C: (a–g) images obtained 0, 1.5, 3.5, 4, 5, 10 and 50 min, respectively, after the mixing of a dispersion of GMV and a solution of precursor V* (taken from [36]).

In a different manner, a self-reproducing vesicle system was developed by designing new amphiphilic molecules [36,37]. The designed precursor of the membrane molecule (V*) is a bolaamphiphile (a polar head group is connected at one end of a tail by imide bonding) and is thus easily hydrolyzed to a membrane molecule (V) and electrolyte (E) through the aid of the catalyst, C, as shown in Figure 6A. When the precursor V* is added to a suspension of vesicles composed of the membrane molecule, V, and catalyst, C, V* is hydrolyzed in the vesicular membrane. The mother vesicle undergoes division using the generated membrane molecule, V, as shown in Figure 6B. These novel observations suggest the potential of membrane molecules to cause the self-reproduction of vesicles, although the physical basis is not clear. It is worthwhile to note that Sugawara’s group developed an advanced model protocell where the self-reproducing vesicle system is coupled with the amplification of DNA [38]. The interplay between DNA and the vesicular membrane accelerated the division of the vesicles.

In terms of membrane free energy, the self-reproduction is not straightforward [29]. During the shape deformation, a spherical vesicle should transform to a limiting shape where two spheres are connected by a very thin neck; then, the neck breaks. To achieve the deformation into the limiting shape, the reduced volume and the reduced intrinsic area difference are changed to a point on the line L^pear^ in Figure 2 through the incorporation of new membrane molecules into the vesicle and through volume changes caused by the water flow through the membrane. A geometrical requirement for the division should satisfy the condition $T_{d}L_{p}\kappa C_{0}^{4}\geq 1.85$, where *L*_p_ is the membrane hydraulic permeability and *T*_d_ is the time taken for the membrane to double its area [39,40]. The sizes of the mother and daughter vesicles depend on the value of $T_{d}L_{p}\kappa C_{0}^{4}$. At smaller values, a growing vesicle will exhibit shapes that cannot lead to self-reproduction. Another important problem is the pinching off a daughter vesicle from the mother vesicle. Generally, single-component vesicles show the shape deformation from sphere to the limiting shape, but no fission event occurs [41], because the vesicle division has an elastic energy penalty (Gaussian energy cost) due to the Gauss–Bonnet theorem in Equation (2). Thus, we have to design a protocell system that satisfies these physical requirements.

In addition, the ingredients should be transported from the external solution to the vesicle and converted to membrane molecules through the aid of catalysts. In order to develop autonomous protocells, the metabolic system might be autocatalytic, where membrane molecules themselves act as catalysts in reaction networks of metabolic pathways [42,43]. Thus, the vesicles should control the traffic of reactants and products, which includes the encapsulation of external ingredients. The coupling between the self-reproduction of a vesicle and an autocatalytic system is a milestone in developing protocells.

## 2. Amphiphilic Molecules

### 2.1. Architecture of Amphiphilic Molecules

**Figure 7:**
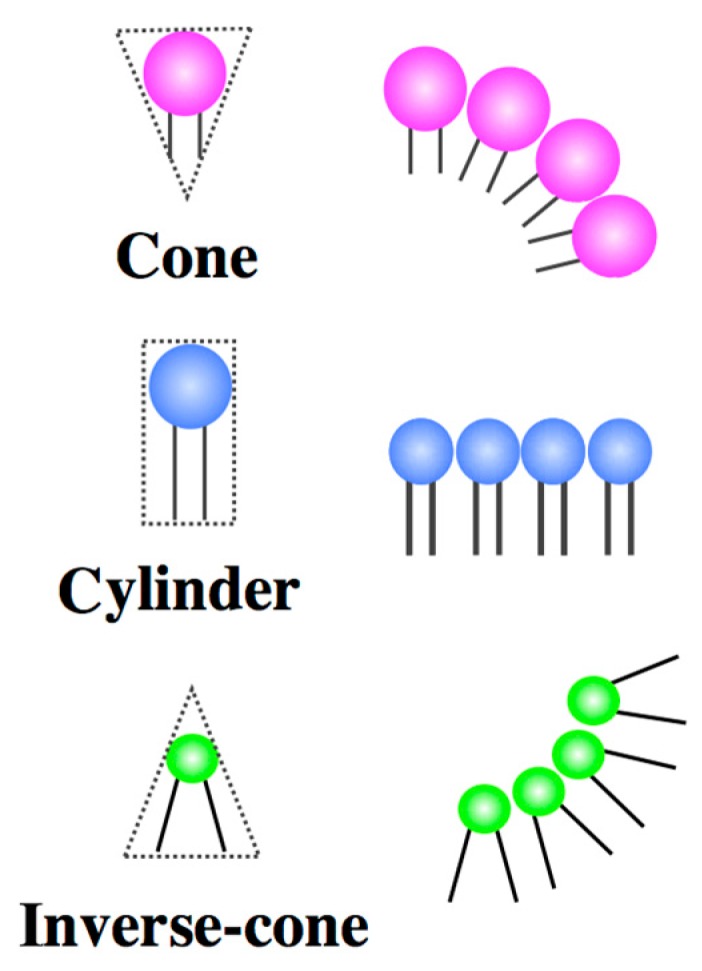
Schematic representations of cone-, cylinder- and inverse-cone-shaped lipids.

In present cellular life, the deformations relevant to biological functions are controlled by specific proteins. The proteins contribute to the local membrane curvatures by interacting with lipids [44]. These sophisticated proteins are, however, the result of evolution over a period of several billion years. In the early stage of the protocell era, amphiphilic molecules may have played the roles of the proteins. In this review, we demonstrate how amphiphilic molecules can influence the local membrane curvature relevant to cellular life. The key concept is a coupling between the molecular shape and the distribution of amphiphilic molecules. From a geometrical point of view, the shape of amphiphilic molecules can be classified into three types (spontaneous curvatures): a cone, a cylinder and an inverse-cone shape (Figure 7) [3]. If the amphiphilic molecule has small hydrocarbon chains and a large headgroup (e.g., the cone shape), the monolayer has a tendency to bend toward the side of the tail. Similarly, an amphiphilic molecule having bulky hydrocarbon chains and a small headgroup (e.g., the inverse-cone shape) tends to bend the monolayer toward the side of the headgroups. An amphiphilic molecule that prefers to form a flat monolayer is referred to as a cylinder-shaped molecule. In the case of the flat bilayer, such geometrical preference is cancelled due to the symmetry of the bilayer. In the curved bilayer, however, the asymmetric amphiphilic molecules prefer to partition in a leaflet having the same bending direction. For example, inverse-cone shaped lipids prefer to partition in the inner leaflet of a spherical vesicle, and the lipids in the outer leaflet are highly frustrated. This geometrical frustration causes shape deformations.

### 2.2. Binary Vesicle

**Figure 8:**
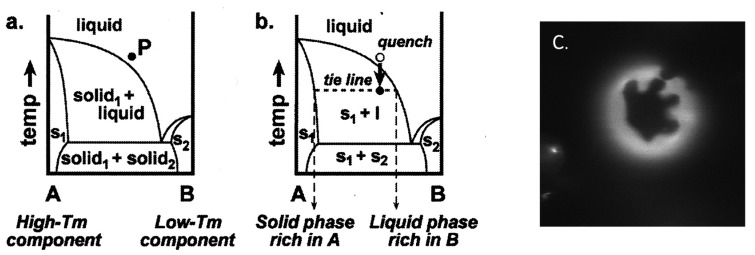
Binary phase diagrams containing coexisting solid and liquid phases. (**a**) At high temperatures, Components A and B mix completely in one uniform liquid phase. An arbitrary mixture of A and B is shown at Point P. (**b**) When the temperature is quenched, the phase boundary is crossed, and the system separates along the tie-line into a solid phase rich in Component A and a liquid phase rich in Component B (taken from [46]). (**c**) The fluorescence micrograph for phase separated binary GUV composed of 1,2-dipalmitoyl-*sn*-glycero-3-phosphoethanolamine (DPPE) (rich in solid phase) and 1,2-dioleoyl-*sn*-glycero-3-phosphocholine (DOPC) (rich in liquid phase). s, solid.

Binary vesicles composed of two types of lipids having different geometries are good model systems to demonstrate the shape deformation caused by geometrical frustration. Phospholipids are composed of a phosphate-based hydrophilic moiety and an acyl chain-based hydrophobic moiety. The acyl chains of the lipids exhibit a main chain transition, *i.e.*, order-disorder transition of acyl chains, when the temperature changes. The melting temperature depends on the length and/or the chemical structure (double bonds and side chains) of the acyl chain. It should be noted that the cross-section area of the lipid increases upon chain melting. For 1,2-dipalmitoyl-*sn*-glycero-3-phosphocholine (DPPC), having a chain melting temperature (*T*_m_) of 41 °C, the cross-section area increases from 47.9 Å^2^ (20 °C) to 64 Å^2^ (50 °C) [45]. A schematic phase diagram of lipid membranes composed of high *T*_m_ lipids and low *T*_m_ lipids is shown in Figure 8a [46]. At high temperatures, a mixture of the two components in one uniform liquid phase is observed. Between high *T*_m_ and low *T*_m_, the system separates into coexisting solid (s) and liquid (l) phases (Figure 8b). The phase separation is visualized using fluorescence dyes that prefer to localize in the liquid phase. A fluorescence micrograph of a phase separated, binary GUV composed of 1,2-dipalmitoyl-*sn*-glycero-3-phosphoethanolamine (DPPE, *T*_m_ = 63 °C) and 1,2-dioleoyl-*sn*-glycero-3-phosphocholine (DOPC, *T*_m_ = −20 °C) is shown in Figure 8c. In the image, the dark domain (solid phase) is strongly enriched in high *T*_m_ lipids (DPPE), whereas the bright domain (liquid phase) is rich in low *T*_m_ lipids (DOPC). The domains grow through a diffusion and coalescence mechanism. The liquid domains have a circular shape due to the line tension, whereas the solid domains prefer to form a diffusion-limited aggregation shape (dendritic pattern) [47], as shown in Figure 8c. Two solid phases can be immiscible at low temperatures, as shown in Figure 8a. Using the phase separation into solid and liquid phases, we can laterally segregate the lipids having different shapes.

## 3. Functions of the Protocell Reproduced by Binary Vesicles

We demonstrate that the deformations relevant to the protocell can be reproduced by mixing two types of lipids having different geometries. The main chain transition temperature of each lipid is adjusted by choosing appropriate acyl chains (length and double bonds). When we decrease the temperature of the binary GUV from the homogeneous one-phase region, the binary GUV undergoes phase separation, and the lipids that have an asymmetric geometry are segregated on the vesicle. To release the frustration due to the geometrical mismatch between the membrane curvature and the lipid geometry, the GUVs undergo a shape deformation depending on the shape of the lipids. Here, we focus on two types of GUV deformation relevant to the membrane trafficking, adhesion [48] and pore formation [49]. In addition, we show that the binary vesicles have a potential to undergo self-reproduction [50].

### 3.1. Adhesion

To establish metabolism in protocells, ingredients must be supplied to the reaction network inside of the protocell from the external medium, and unnecessary side-products must be excreted from the cell. Because the cell membrane is almost impermeable to ions and macromolecules, present cells have developed sophisticated vesicular transportation systems, endocytosis and exocytosis, that are supported by transmembrane proteins and peptides [51]. The adhesion of vesicles plays a central role in vesicular transportation (exocytosis). In the present cell, the highly selective interactions leading to cell adhesion are mediated by a variety of specific receptors that are embedded in the cell membranes. The physical basis of cell adhesion is described in review articles [52,53].

**Figure 9:**
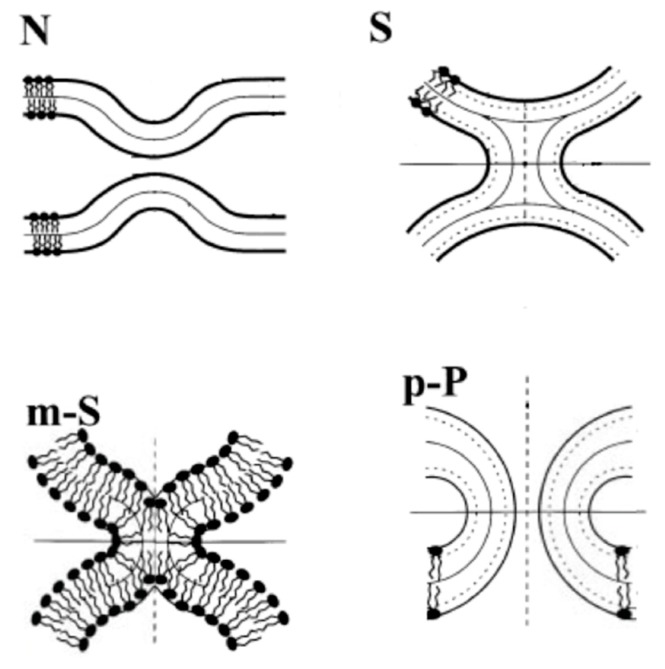
Illustrations of the intermediate structures of membrane fusion: nipples (N), stalk (S), modified stalk (m*-*S) and prepore (p-P). The bold solid lines are drawn along the polar head groups of the lipid molecules (taken from [54]).

A model fusion pathway presented using an energetic calculation is described in Figure 9 [54]. The fusion starts from the protrusion of two apposed membranes that form contact site “nipples” (N). The bending energy calculation reveals that the nipples decrease the bending energy cost for the formation of the “stalk” (S) intermediate structure, where the two apposed monolayers merge (hemifusion) [55]. Because the stalk has a hydrophobic void with prohibitively high energy, a transformation of the stalk to a modified stalk (m-S) might take place by small movements of the lipids. This modified stalk develops into the next intermediate, the prepore (p-P), to form the fusion pore. The fusion pore expands, and fusion occurs. Thus, hemifusion is a relevant adhesion in vesicular transportation. Hereafter, we focus on the hemifusion of vesicles.

**Figure 10:**
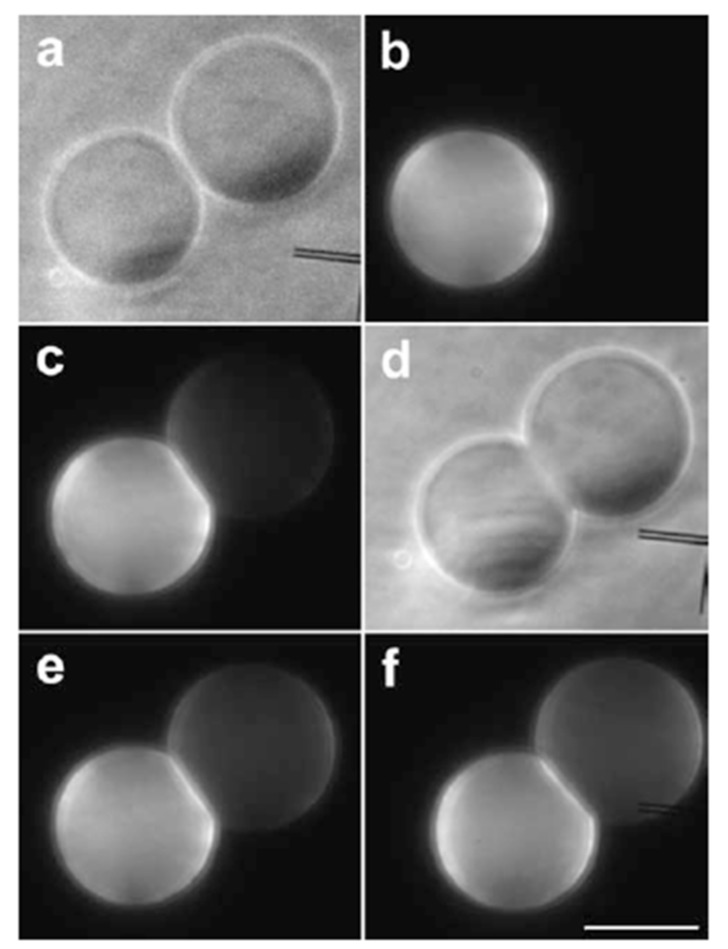
Two tangent vesicles observed by phase contrast microscopy (**a**) and epi-fluorescence (**b**); one of the vesicles is functionalized by adenosine lipids and labeled by 1,2-dioleoyl-*sn-*glycero-3-phosphoethanolamine-N-(lissamine rhodamine B sulfonyl) (RhPE), and the other one is functionalized by thymidine lipids. Just after aggregation of the two vesicles, as attested by membrane deformation in the contact area (**d**), a fluorescent signal appeared on the non-labeled vesicle (**c**), denoting a lipid mixing. Fluorescent lipids carry on their redistribution after 3 min (**e**), until they reach equilibrium after 6 min (**f**). The bar is 20 μm (taken from [56]).

The formation of the hemifusion intermediate during the fusion process is confirmed by a fluorescent lipid transfer experiment where fluorescent labeled lipids transfer from a labeled vesicle to a non-labeled vesicle through the hemifused membrane [56]. In this experiment, an attractive interaction between vesicles was introduced by adding functionalized lipids bearing the DNA bases, thymidine or adenosine, as headgroups (H-bond between nucleosides). This attractive interaction keeps the two giant vesicles at an intermembrane distance of 1 nm (nipples). For the lipid transfer experiment, two types of vesicles predominantly composed of DOPC were prepared. The first type contained adenosine lipids and fluorescent labeled lipids, 1,2-dioleoyl-*sn-*glycero-3-phosphoethanolamine-N-(lissamine rhodamine B sulfonyl) (RhPE). The second type bore thymidine lipids and had no fluorescent lipids. When the two vesicles were brought into close proximity, the vesicles adhered spontaneously (Figure 10a,b). Immediately after the adhesion, a fluorescence signal was observed on the non-labeled vesicle (Figure 10c). During a few minutes, the fluorescence intensity on the thymidine-bearing vesicle increased, while the fluorescence intensity decreased on the adenosine-bearing vesicle (Figure 10e,f), indicating the formation of the hemifusion state. In this experiment, several adhering vesicles proceeded to fusion (~5%), and the remaining adhering vesicles reached equilibrium.

**Figure 11:**
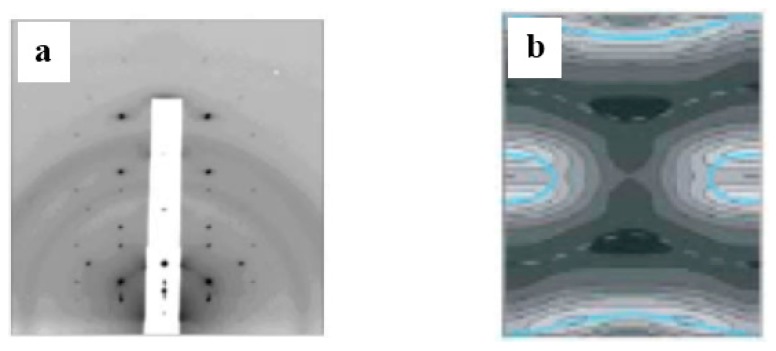
(**a**) Diffraction pattern of a rhombohedral structure (space group $R\bar{3}$); (**b**) the constructed electron density map shows a stalk structure (taken from [57]).

A geometrical consideration suggests that the inverse-cone-shaped lipids tend to promote the formation of the stalk. The formation of the stalk by the inverse-cone-shaped lipids was demonstrated using an X-ray diffraction technique [57]. The inverse-cone-shaped lipids (1,2-diphytanoyl-*sn*-glycero-3-phosphocholine: DPhPC) form the stacked bilayer, called the lamellar phase, when the inter-membrane distance on a clean and flat substrate is sufficient. For decreases in the inter-membrane distance, a scattering pattern having a rhombohedral space group was observed, as shown in Figure 11a. The three-dimensional electron density map constructed from the diffraction pattern (Figure 11b) shows that the two apposed monolayers merged and bent into an hourglass shape, which is exactly what has been modeled as the stalk.

**Figure 12:**
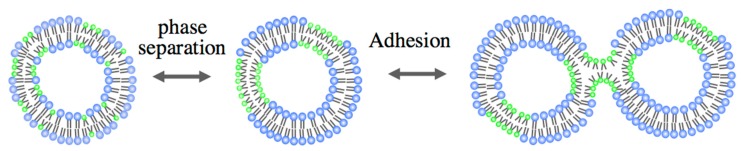
Schematic representation of the hemifusion of binary GUVs composed of inverse-cone-shaped lipids (green) and cylinder-shaped lipids (blue).

Here, we demonstrate the hemifusion of vesicles using binary GUVs composed of inverse-cone- and cylinder-shaped lipids [48]. Through phase separation, the binary vesicles form domains that are rich in the inverse-cone shape lipids. In the outer leaflet of the domains, the inverse-cone-shaped lipids are in a highly stressed state due to the geometrical mismatch. When the two phase-separated vesicles are brought into contact, the two apposed monolayers in the domains might merge and develop into the stalk [54,55] using the spontaneous curvature, as shown in Figure 12.

**Figure 13:**
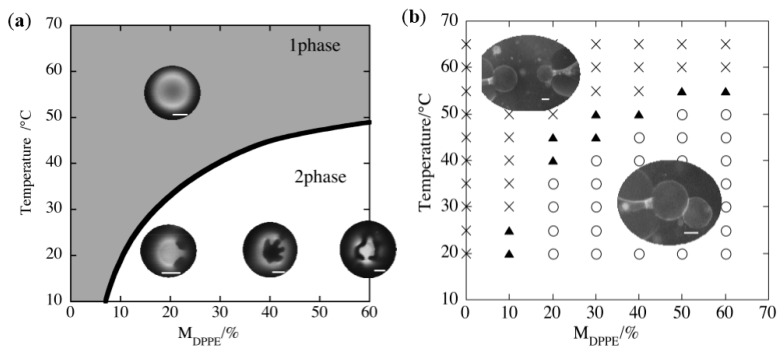
(**a**) Phase diagram of DPPE/DOPC binary GUV. Fluorescence micrograph images in the figures show the appearance of a vesicle at the one phase and two phase region. *M*_DPPE_ is the mole fraction of DPPE. (**b**) Adhesion diagram of DPPE/DOPC binary GUV. Circles, crosses and triangles indicate adhesion, non-adhesion and boundary states, respectively (taken from [48]).

The hemifusion of GUVs induced by the inverse-cone-shaped lipid was realized using binary GUV composed of DPPE (inverse-cone-shaped lipid, *T*_m_ = 62 °C) and DOPC (cylinder-shaped lipid, *T*_m_ = −20 °C). The phase diagram is shown in Figure 13a, where the binary membrane with a DPPE mole fraction *M*_DPPE_ >60% could not form GUVs due to the geometry of DPPE. Based on the phase diagram, the adhesion of the binary GUVs was examined by attaching two GUVs using a micro-manipulation technique. The obtained adhesion diagram for the DPPE/DOPC binary GUV is shown in Figure 13b, where circles indicate that the two GUVs adhere to one another in response to the contact, while cross symbols mean that the GUVs do not exhibit adhesion and triangles indicate the boundary region. The adhesion/non-adhesion boundary in Figure 13b agrees well with the immiscible boundary of the DPPE/DPPC binary vesicles in Figure 13a. In addition, when the temperature is increased so that one homogeneous phase region is present, the adhering vesicles separate spontaneously, indicating that the phase separation is responsible for the observed adhesion. A similar adhesion behavior was also confirmed for binary GUVs composed of DPhPC (inverse-cone-shaped lipid, *T*_m_ < −120 °C) and DPPC (cylinder-shaped lipid, *T*_m_ = 41 °C).

**Figure 14:**
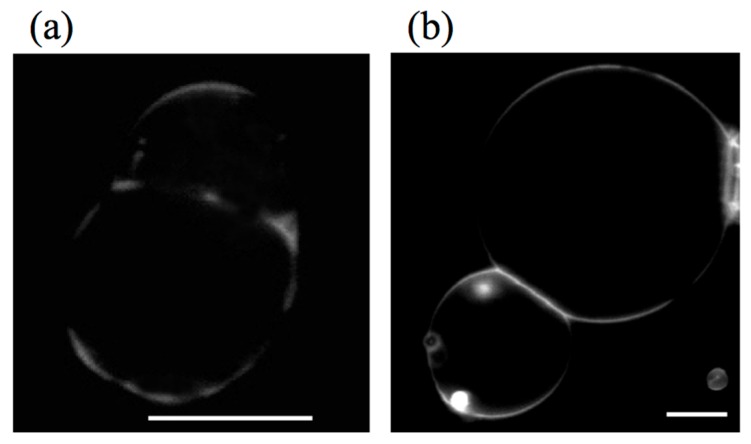
Fluorescence images of (**a**) adhering DPPE/DOPC binary GUVs and (**b**) 1,2-diphytanoyl-*sn*-glycero-3-phosphocholine (DPhPC)/DPPC binary GUVs. Scale bars indicate 10 μm (taken from [48]).

Figure 14 shows images of adhering binary GUV: (a) DPPE/DOPC and (b) DPhPC/DPPC. The GUVs were dyed with Texas red-1,2-dihexadecanoyl-*sn*-glycero-3-phosphoethanolamine (TR-DHPE), and TR-DHPE is localized in the liquid phases. For DPPE/DOPC binary GUVs, the GUVs adhere to one another through the dark region, namely the DPPE-rich domains. Similarly, the adhering region of DPhPC/DPPC binary GUVs shows an intense line, indicating that the adhesion takes place through the DPhPC-rich region. These images clearly show that the GUVs adhere to one another through the domains that are rich in the inverse-cone-shaped lipids.

**Figure 15:**
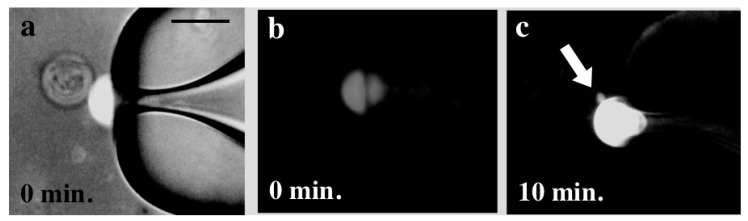
Two adhering GUVs composed of DPPE/DOPC (= 6/4) in a fluorescent lipid transfer experiment, (**a**) phase contrast + fluorescence image and (**b**) fluorescence image for adhering GUVs just after they contact. The bright GUV is a fluorescently labeled GUV, and the non-labeled GUV is positioned at the left side of the labeled GUV (detected by the phase contrast image in (a) and not seen in (b)). After 10 min, a fluorescence signal appears in the non-labeled GUV in the neighborhood of the contacting area (indicated by an arrow) in the fluorescence image (**c**) (taken from [48]).

The adhesion through the stalk intermediate was confirmed by a fluorescence lipid transfer experiment, as shown in Figure 15. Here, two types of binary vesicles composed of DOPC and DPPE were prepared. The first type contained the fluorescently labeled lipids, TR-DHPE, and the second type had no fluorescence lipids. Two phase separating vesicles of different types were brought into contact for adhesion using micro-manipulation (Figure 15a, phase contrast and fluorescence overlapped image). Just after the adhesion, the labeled vesicle showed a fluorescence signal, whereas the non-labeled vesicle could not be detected by a fluorescence microscope observation, as shown in Figure 15b. Slightly after the adhesion (several minutes), a fluorescence signal was observed in the neighborhood of the contact area on the non-labeled vesicle, as shown by the arrow in Figure 15c, indicating the hemifusion through the inverse-cone lipid domains. It should be noted that the lipid transfer was observed in a limited region due to the irregular domain shape. The formation of the stalk leads to a decrease in the bending energy and line energy in the adhering monolayers, which stabilizes the hemifusion state. Unfortunately, it is difficult to induce a transformation of this hemifusion state to the fusion state due to the stability of the intermediate. Precise control of the lipid shape might be needed to achieve fusion.

### 3.2. Pore Formation

Pore formation is an important membrane transport mechanism in the protocell [58]. In living cell systems, pore-forming proteins and peptides are utilized to make a pore in the membrane [59,60].

**Figure 16:**
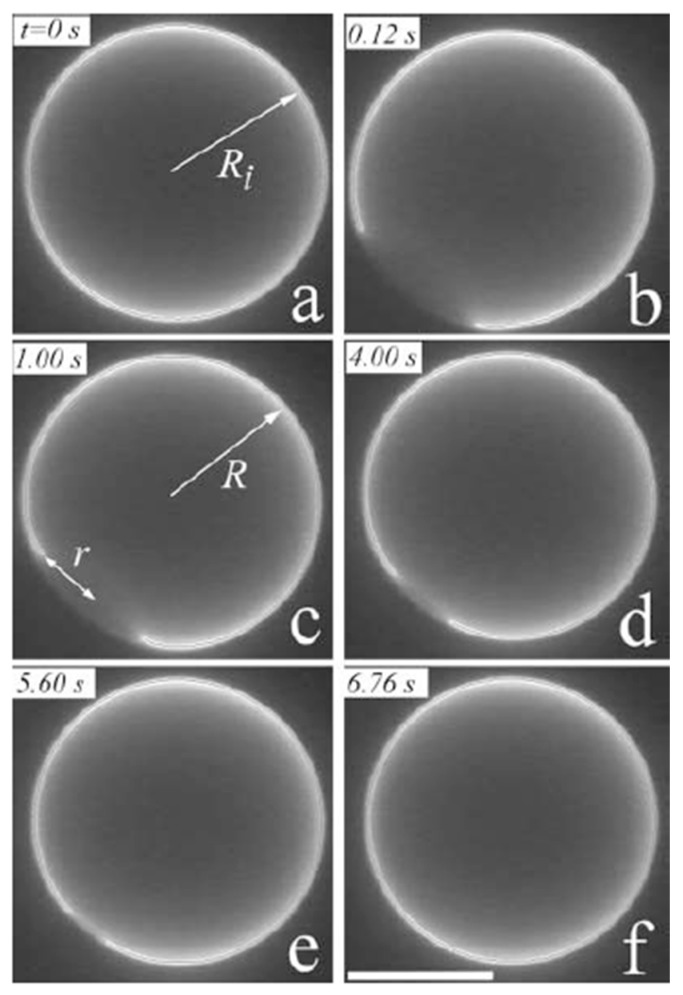
Appearance of a transient pore in a DOPC vesicle. Initially the DOPC vesicle has a spherical shape (a). When the membrane tension reaches a critical value, the vesicle responds with the sudden opening of a pore. The pore size reaches its maximum very rapidly (**b**) and slowly decreases thereafter, until complete resealing (**c**–**f**). The white bar in (f) corresponds to 10 μm (taken from [63]).

Here, we focus on the pore formation in the prebiotic environment, *i.e.*, without peptides and proteins. A simple technique to form the pore in the vesicle is through the control of the membrane tension [61,62,63,64]. Figure 16 shows an example of the pore formation process [63]. When detergents are added to the vesicle suspension (a), the detergents solubilize the membrane, which increases the membrane tension. When the membrane tension reaches a critical value (on the order of a few mN/m), the vesicle responds with the sudden opening of a pore, and simultaneously, the inner solution flows out through the pore (b). Thereafter, the membrane tension decreases rapidly, and the line tension closes the pore (c–f). For the cylinder shape lipids, the line tension is always high, which makes the pore transient.

**Figure 17:**
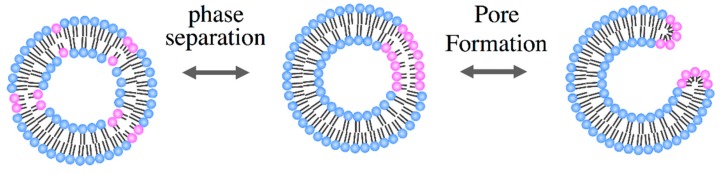
Schematic representation of the pore formation of binary GUVs composed of cone-shaped lipids (red) and cylinder-shaped lipids (blue) (taken from [49]).

**Figure 18:**
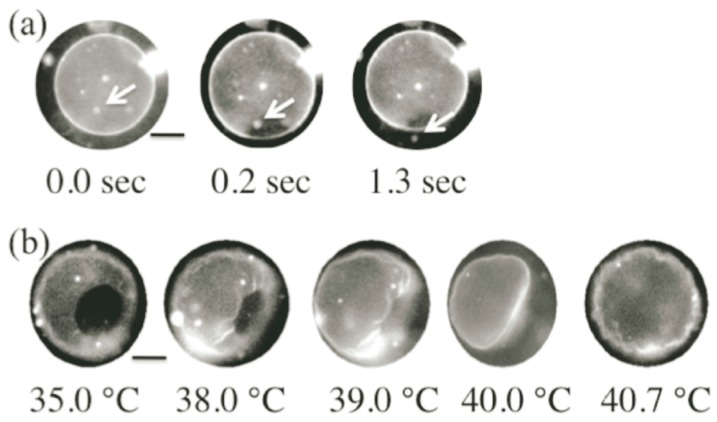
Snapshots of the formation (time evolution at 38.9 °C) (**a**) and closing (temperature dependence) (**b**) of a pore in GUV with DPPC:DHPC = 99:1. When a pore opens, a small vesicle is ejected through the pore, as shown by the arrows in (a). Scale bar is 5 μm (taken from [49]).

We demonstrate stable pore formation using binary GUVs composed of cone- and cylinder-shaped lipids [49]. The binary GUV shows a phase separation between a solid phase that is rich in cylinder-shaped lipids and a liquid phase that is rich in cone-shaped lipids. The segregated cone-shaped lipids might form a cap at the edge of the bilayer due to the geometrical preference, which may stabilize the pore, as shown in Figure 17. The cone-shaped lipid-induced pore formation was realized by using binary GUV composed of 1,2-dihexanoyl-sn-glycero-3-phosphocoline (DHPC: cone-shaped lipid, *T*_m_ = −46 °C) and DPPC (cylinder-shaped lipid, *T*_m_ = 41 °C). In water, DHPC molecules form micelles with a size of *ca.* 2.0 nm [65], indicating that a DHPC molecule has a cone shape with a spontaneous curvature of *ca.* 0.5 nm^−1^. In the one-phase region above the *T*_m_ of DPPC, the binary GUVs showed a spherical shape with radii of 10–30 μm, where both lipids are mixed homogeneously at the molecular scale. For the temperature decreases to below the *T*_m_ of DPPC, the spherical GUVs showed a burst at temperatures below *T*_m_ of DPPC, as shown in Figure 18a. The cross-section area of a DPPC molecule below the *T*_m_ is approximately 78% of that above *T*_m_ [45]. This decrease in the molecular area increases the tension of the vesicle membrane, resulting in membrane fracture. After the burst, a spherical GUV had a single pore, and the inner solution was released from the vesicle through the pore, as shown by the arrow in Figure 18a. The pore was stable below the *T*_m_ of DPPC, because the segregated cone-shaped lipids cap the edge of the bilayer at the rim of the pore, which decreases the line tension at the rim. In contrast, DPPC in the solid state solidifies the main body of the GUV. A unique feature of the pore formation in the binary GUV containing the cone-shaped lipids is that the pore opening and closing could be controlled by the temperature. For increases in the temperature of the GUV with a pore to the one-phase region, the pore started to shrink and closed just below the *T*_m_ of DPPC, as shown in Figure 18b. The GUV recovered its spherical shape as a result of the chain melting. Similar pore formations are observed for other pairs of cylinder-shaped lipids and cone-shaped lipid, *i.e.*, 1,2-distearoyl-sn-glycero-3-phosphocholine (DSPC: *T*_m_ = 54 °C)/DHPC and 1,2-dipentadecanoyl-sn-glycero-3-phosphocholine (15:0 PC: *T*_m_ = 34 °C)/DHPC mixtures, where the pore-opening temperature increases with an increase in the main chain transition temperature [49]. Thus, the coupling between the lipid shape and the main chain transition is responsible for pore formation.

### 3.3. Self-Reproduction of Vesicle

Development of self-reproducing vesicle systems is the indispensable step to achieve autopoietic cycles. Several synthetic biology groups have succeeded in developing self-reproducing vesicle systems, as described in the Introduction [31,32,33,34,35,36,37,38], although we are still far from understanding the mechanisms. To address the mechanism, it is desirable to develop model vesicle systems that are capable of undergoing division in a controlled manner.

**Figure 19:**
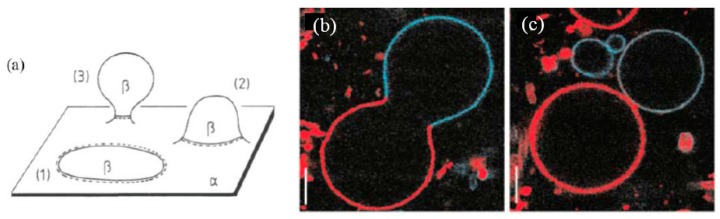
(**a**) Budding of membrane domain β embedded in the membrane matrix α (taken from [66]); budding (**b**) and complete budding (**c**) of phase separated vesicle composed of sphingomyelin, DOPC and cholesterol. The phase separation was visualized using perylene (blue) in the L_o_ phases and rhodamine-DPPE (red) in the L_d_ phases. Scale bars, 5 μm (taken from [68]).

A simple route to produce budding and fission events on the vesicle is to use the phase separation of the binary vesicle [66,67,68]. Through phase separation, domains having different lipid compositions appear on the vesicle (β phase in the α phase: Figure 19a), which results in a line tension at the domain boundary. A competition between the bending energy and the line energy causes budding. When the bending energy governs the system, the membrane prefers a flat geometry (Figure 19a-1). In contrast, when the line energy is dominant, the vesicle forms a bud to decrease the edge length of the domain, and finally, the bud domain forms a separated vesicle (complete budding), where the line energy disappears (Figure 19a-2,a-3). The budding of a phase separated vesicle is shown in Figure 19b,c. The vesicle is composed of sphingomyelin, DOPC and cholesterol. Through phase separation, sphingomyelin and cholesterol enrich in a liquid phase with short-range order (L_o_), and DOPC prefers a disordered liquid (L_d_) phase. The phase separation was visualized using perylene (blue) in the L_o_ phases and rhodamine-DPPE (red) in the L_d_ phases. In this budding mechanism, however, the composition of the daughter vesicle is different from that of the mother vesicle, *i.e.*, it is not recursive.

Recently, Sakuma *et al.* [50] developed a temperature-controlled cyclic self-reproducing vesicle system without feeding. The cyclic self-reproduction means that the mother vesicle produces identical daughter vesicles through a cyclical change in an external condition. In the second cycle, the mother and the daughter vesicles produce the second daughter vesicle and the granddaughter vesicle, respectively. The vesicle is composed of cylinder-shaped lipids with a high melting *T*_m_ (DPPC, *T*_m_ = 41 °C) and inverse-cone-shaped lipids with a low *T*_m_, (1,2-dilauroyl-sn-glycero-3-phosphoethanolamine: DLPE, *T*_m_ = 29 °C). Lipids with a phosphoethanolamine head group (PE lipids) are frequently observed during a topological change of the cellular membrane, *i.e.*, fission and fusion events [69,70], indicating that the PE lipids encourage topological transitions.

**Figure 20:**
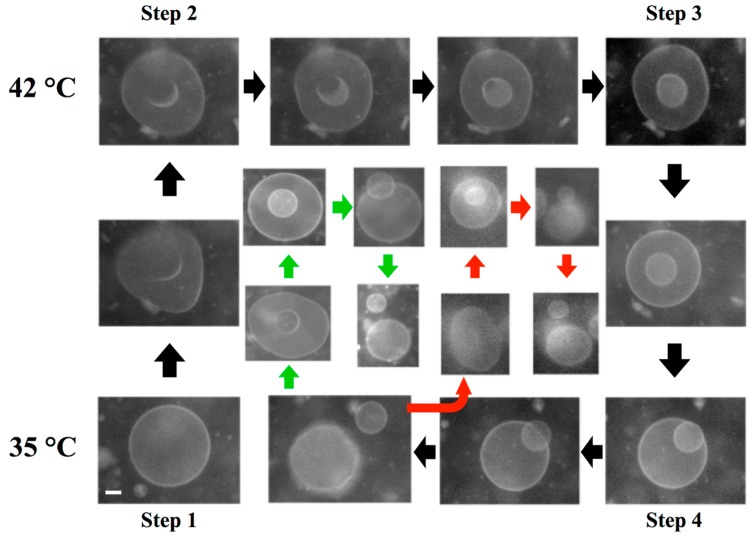
A series of snapshots of the birthing process observed in the binary vesicle composed of 1,2-dilauroyl-*sn*-glycero-3-phosphoethanolamine (DLPE)/DPPC = 3/7. The birthing cycle consists of four steps: (1) sphere to stomatocyte deformation; (2) formation of the inclusion vesicle; (3) birthing of the daughter vesicle through a pore; and (4) recovery of the spherical vesicle by closing the pore. The green and red pathways show the birthing of the second daughter vesicle and the granddaughter vesicle, respectively. The scale bar indicates 5 μm.

A unique feature of the model system is that it can reproduce the birthing and the budding pathways. The observed birthing pathway in the binary GUVs with a composition of DLPE/DPPC = 3/7 is shown in Figure 20. The experiment was started from a spherical vesicle at 35 °C, below the *T*_m_ of DPPC (Step 1). By increasing the temperature above the *T*_m_ (42 °C), chain melting took place, which produced the excess area. Using the excess area, the spherical GUV deformed to a stomatocyte shape (Step 2). As time elapsed, the stomatocyte vesicle spontaneously formed an inclusion vesicle inside the mother vesicle by pinching off the invagination neck (Step 3). When the temperature was decreased below the *T*_m_ of DPPC (35 °C), the surface area of the mother vesicle decreased due to chain ordering. This change resulted in an increase in the membrane tension of the mother vesicle. To release the tension, the mother vesicle formed a single pore, and the inclusion vesicle was discharged through the pore, *i.e.*, birthing of the daughter vesicle (Step 4). After the birthing, the pore was immediately resealed due to the line tension, and the mother GUV recovered a spherical shape, although the resulting GUV had a smaller size as the original GUV. Interestingly, when the temperature was again increased above the *T*_m_, the recovered spherical mother GUVs formed inclusion vesicles, and then, a second daughter vesicle was born when the temperature decreased below the *T*_m_ (Figure 20, green pathway). In addition, the daughter vesicle followed the same birthing pathway and produced granddaughter vesicles (Figure 20, red pathway). This result indicates that the birthing ability is maintained in the next generation (sometimes fourth- or fifth-generation vesicles).

**Figure 21:**
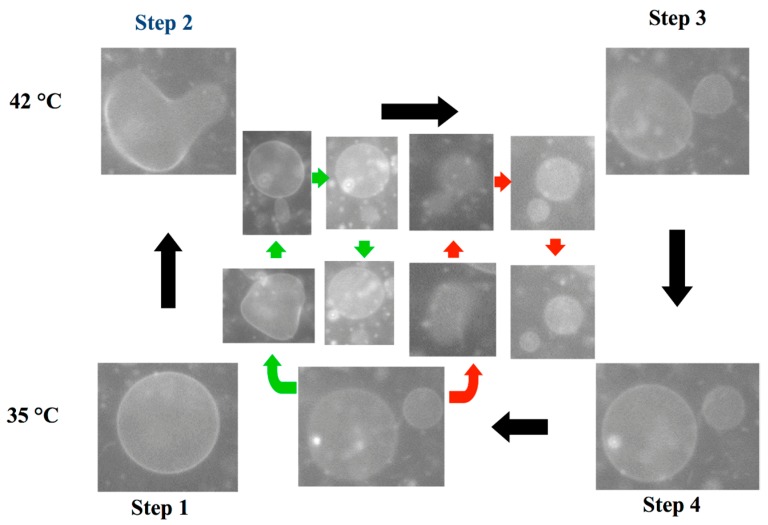
A series of snapshots of the budding process of a binary vesicle composed of DLPE/DPPC = 2/8. The budding cycle consists of four steps: (1) sphere; (2) budding; (3) complete budding; and (4) recovery of the spherical shape. The green and red pathways show the budding of the second daughter vesicle and the granddaughter vesicle, respectively. The scale bar indicates 5 μm.

Through temperature changes, DPPC lipids undergo the main chain transition, *i.e.*, change of the cross-section area, whereas DLPE retains a constant area. This asymmetry is the key to the observed self-reproduction. Thus, the control parameter is the composition of the binary vesicle. When the composition of the GUV was changed to DLPE/DPPC = 2/8, the GUVs showed the budding pathways depicted in Figure 21. Again, the experiment was started from a spherical vesicle at 35 °C (Step 1). By increasing the temperature of the GUV above the *T*_m_, GUVs showed outer budding deformations (Step 2), and then, the buds were completely pinched off; *i.e.*, daughter vesicles were produced by the budding pathway (Step 3). Through decreases in the temperature, the mother and the daughter vesicles recovered a spherical shape (Step 4). During the second temperature cycling, the mother GUV repeated the budding pathway and produced a second daughter vesicle (green pathway), and the first daughter vesicle produced a granddaughter vesicle (red pathway) by the budding mechanism. Again, the budding ability was maintained in the next generation. During multi-temperature cycling, the vesicles consistently divided through the budding mechanism, not via the birthing pathway, which suggests conservation of the composition of the binary GUV. It should be noted that in the self-reproduction of vesicles, membrane molecules that are supplied from the metabolic pathway or the external environment are incorporated into the vesicle membrane, which increases the membrane area. Using this excess area, vesicles deform to the limiting shape and then produce a daughter vesicle. Unfortunately, at present, it is very difficult to couple the model vesicle with the metabolic pathway. Thus, in this model system, the change in the cross-section area due to the main chain transition of the lipid is used to increase the membrane area. The shape deformation pathway observed in this model system is identical with that observed in the reported self-reproduction system [31,32]. Through decreases in the temperature, the area of the model vesicle returns to the initial area, *i.e.*, the system is cyclic. Thus, this model system captures the heart of self-reproduction.

Here, we consider two important aspects to achieve vesicle division, the shape deformation pathway and the breaking of the neck. To achieve the shape deformation from a sphere to a limiting shape (budding pathway), the reduced intrinsic area difference, $\Delta a=\Delta A/8\pi dR_{s}$, has to increase from one to $(R_{m}+R_{d})/\sqrt{{R_{m}}^{2}+{R_{d}}^{2}}$, where *R*_m_ and *R*_d_ are the radii of mother and daughter vesicles, respectively. One factor of this increase in the area difference is attributed to the introduction of the inverse-cone-shaped lipid, DLPE. The spontaneous curvature of DLPE, $H_{sp}^{PE}≃$ −0.3 nm^−1^ [71], causes an asymmetric distribution in the bilayer, *i.e.*, DLPE molecules prefer the inner leaflet of a spherical vesicle. At the initial stage (Step 1 in Figure 21), the binary vesicle has a spherical shape. Using cross-section areas of a DLPE lipid, *a*_DPLE_, and a DPPC lipid in the solid state, *a*_DPPC_, we can express the intrinsic membrane areas of inner and outer leaflets by $A_{0}^{in}=(a_{DLPE}\phi^{in}+a_{DPPC}(1-\phi^{in}))n^{in}=4\pi R_{in}^{2}$ and $A_{0}^{out}=(a_{DLPE}\phi^{out}+a_{DPPC}(1-\phi^{out}))n^{out}=4\pi {\left(R_{out}+d\right)}^{2}$, respectively, where the molar fraction of DLPE in the inner and the outer leaflets is expressed by $\phi^{in}=n_{DLPE}^{in}/n^{in}$ and $\phi^{out}=n_{DLPE}^{out}/n^{out}$, respectively ($n_{DLPE}^{i}$: the number of DLPE lipids in the *i* leaflet; *i* = in or out). For simplicity, we assume *a*_DLPE_ = *a*_DPPC_ = *a*. Then, the reduced volume and the intrinsic area difference of the initial spherical vesicle are expressed by *v* = 1 and $\Delta a_{0}=1$, respectively. When the temperature increases above the *T*_m_ of DPPC (Step 2), chain melting of DPPC takes place, which increases the cross-section area of a DPPC lipid by approximately 40%, *i.e.*, *a*→1.4*a* [45]. Then, the surface area of the GUV increases, while the vesicle volume remains constant. Using the excess area, the GUV deforms to a pear shape. A simple geometrical calculation shows that through chain melting, the reduced volume decreases to $\nu ={\left(1.4-0.4\phi^{in}\right)}^{-3/2}$ and the intrinsic area difference changes to $\Delta A_{0}/(8\pi dR_{in})=1.4-0.4\phi +(R_{in}/d)(\phi^{in}-\phi )$, where ϕ is the average molar fraction of DLPE. The change in the intrinsic area difference strongly depends on the asymmetric transversal distribution of DLPE in the bilayer, ϕ^in^ − ϕ, because the GUV has a very large value of *R*_in_/*d* ~ 2000. Thus, a very small asymmetric transversal distribution of PE lipids in the bilayer governs the deformation pathway.

Another important event in the self-reproduction pathway is the breaking of the narrow neck in the limiting shape vesicle. For a single component vesicle, the neck is stable, and vesicle fission is usually observed in multi-component vesicle [50,67]. Because the fission involves a topological change, the coupling of the local lipid composition to the Gaussian curvature is important for the fission of the vesicle. Chen *et al.* have shown that the coupling of the local lipid composition to the Gaussian curvature can destabilize the narrow neck in the budded state of lipid vesicles [72]. Depending on the molecular geometry of the minor component lipids, this coupling can reduce the Gaussian rigidity, and fission can be enhanced. This possibility is a plausible explanation for the breaking of the neck, although the localization of PE lipids at the regions with large positive Gaussian curvature has not been demonstrated experimentally.

## 4. Conclusions

An important point to consider for the development of protocells is whether the functions relevant to cellular life, such as the membrane trafficking and self-reproduction of the vesicle, were realized before the emergence of proteins. If so, what mechanisms govern the functions? Here, we show that when the molecular geometry is coupled to the main chain transition of lipids, vesicles undergo unique deformations that are relevant to protocell functions. It is plausible that in the prebiotic environment, the vesicles were composed of various types of amphiphilic molecules. The amphiphilic molecules that produce the shape deformations can be classified as cylinder-, cone- and inverse-cone-shaped geometries. A multi-component membrane composed of amphiphilic molecules having different geometries controls the local membrane curvature by lateral and transverse segregation in the bilayer. External stimuli, such as thermal, optical [64,73] and chemical stimuli [74,75], trigger the shape deformation. These primitive functionalities might be integrated into more sophisticated autonomous control systems, *i.e.*, genes and proteins, although this principle of controlling membrane curvature is still present in modern cellular systems, such as for lipid sorting [71,76,77,78,79].
